# The Coagulopathy of Acute Promyelocytic Leukemia: An Updated Review of Pathophysiology, Risk Stratification, and Clinical Management

**DOI:** 10.3390/cancers15133477

**Published:** 2023-07-03

**Authors:** Jack Hermsen, Bryan Hambley

**Affiliations:** 1 University of Cincinnati College of Medicine, Cincinnati, OH 45267, USA; 2 Division of Hematology/Oncology, Department of Internal Medicine, University of Cincinnati, 3125 Eden Ave, Cincinnati, OH 45267, USA

**Keywords:** acute promyelocytic leukemia, all-trans retinoic acid, arsenic trioxide, coagulopathy, hemorrhage, thrombosis

## Abstract

**Simple Summary:**

Outcomes for patients with acute promyelocytic leukemia (APL) have improved over the past 40 years due to the development of targeted treatment regimens and an expanded understanding of the unique complications arising from both the disease and its treatments. Despite these advances, clinically significant bleeding and/or thrombosis in the first 30 days of treatment remain the most common causes of morbidity and mortality. Moreover, there is a lack of large clinical trials investigating approaches for the prevention and treatment of these complications. This review aims to provide a comprehensive overview and update on the coagulopathy associated with APL, compile current best practices for optimal management, and establish areas of potential future investigation to further improve outcomes for patients with APL.

**Abstract:**

Acute promyelocytic leukemia (APL) has a well-established mechanism and a long-term prognosis that exceeds that of any other acute leukemia. These improving outcomes are due, in part, to all-trans retinoic acid (ATRA) and arsenic trioxide (ATO), two targeted and highly active agents in this disease. However, there remains a considerable morbidity and mortality risk in APL secondary to clinically significant hemorrhagic and/or thrombotic events. Prevention and treatment of these coagulopathic complications remain significant impediments to further progress in optimizing outcomes for patients with APL. Moreover, the relative rarity of APL hinders adequately powered randomized controlled trials for evaluating APL coagulopathy management strategies. This review draws from peer-reviewed works falling between initial descriptions of APL in 1957 and work published prior to January 2023 and provides an updated overview of the pathophysiology of hemorrhagic and thrombotic complications in APL, outlines risk stratification parameters, and compiles current clinical best practices. An improved understanding of the pathophysiologic mechanisms driving hemorrhage and thrombosis along with the completion of well-designed trials of management strategies will assist clinicians in developing interventions that mitigate these devastating complications in an otherwise largely curable disease.

## 1. Introduction

Acute myeloid leukemia (AML) is a hematologic malignancy accounting for approximately 12,000 cases and 9000 deaths annually in the United States [1]. Acute promyelocytic leukemia (APL) is a subtype of AML marked by an abnormal proliferation of promyelocytes and has an incidence of 600–800 cases per year in the United States [2]. APL is often characterized by an aggressive presentation involving a complex coagulopathy resulting in bleeding and/or thrombosis prior to or during initial treatment [3]. The most common type of APL is driven by a characteristic translocation involving the retinoic acid receptor alpha (RARα) gene on chromosome 17 with the promyelocytic leukemia (PML) gene on chromosome 15 leading to clonal promyelocyte expansion [4]. While there are other rare variants of APL, the focus of this review is on APL with PML-RARα as most clinical literature utilizes data from patients with this variant [5].

Mucocutaneous and/or gastrointestinal bleeding is the most common presenting symptom of APL with up to 89% of patients presenting with these complaints [6]. The utilization of all-trans retinoic acid (ATRA) and arsenic trioxide (ATO) has improved outcomes in APL, now with greater than 90% long-term relapse-free survival for those who survive the first 30 days after diagnosis [7,8,9,10]. The most significant contributor to treatment failure is an up to 25% mortality rate within the first 30 days after diagnosis with hemorrhage being the most common etiology [10,11,12,13]. Similarly, a report of 1400 patients diagnosed with APL between 1992 and 2007 demonstrated that while there was an improvement in the three-year survival rate from 54.6% to 70.1%, the early death rate of 17.3% did not significantly decrease throughout the same time frame [12]. Studies of APL in pediatric patients also delineate hemorrhage as the main contributor to early morbidity and mortality [14]. In one study of 736 patients with APL, 37 patients (5%) experienced a hemorrhagic event leading to death, most commonly from intracranial hemorrhage (ICH) or pulmonary hemorrhage [15].

Thrombosis is also a significant contributor to morbidity in APL, with up to 20% of patients with APL experiencing a venous or arterial thrombotic event [16]. These thrombotic events most commonly include deep vein thrombosis (DVT), pulmonary embolism (PE), myocardial infarction (MI), and/or ischemic cerebrovascular events [16,17,18]. The dual risk of hemorrhage and thrombosis underscores the unique coagulopathy of APL.

**Figure 1:**
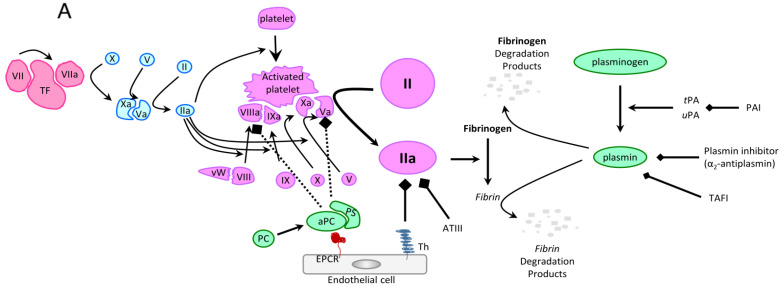

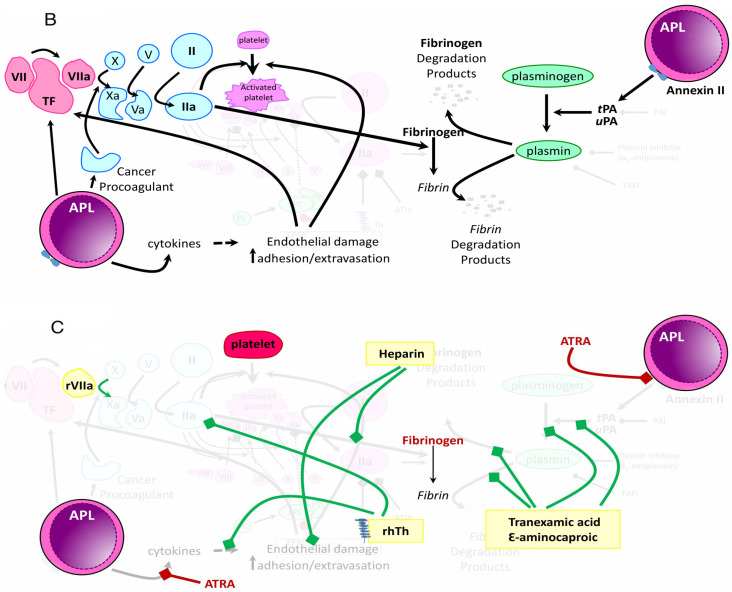
Mechanisms of coagulopathy in APL. (**A**) Normal physiologic balance between hemostasis and thrombosis. Vascular trauma leads to tissue factor (TF) exposure, which promotes the formation of activated factor VII (VIIa). This initiates a cascade starting with activation of factor X, which activates factor V. Activated factor X (Xa) and activated factor V (Va) together cleave prothrombin to thrombin (activated factor II). This process leads to platelet, factor VIII, factor X, and factor V activation and a positive feedback mechanism generating thrombin on the surface of activated platelets. Thrombin cleaves soluble fibrinogen to insoluble fibrin, which enables clot stabilization—an essential aspect of normal hemostasis to reduce hemorrhage [24,25]. This prothrombotic mechanism is counterbalanced via anticoagulant pathways restricting clot formation to the place of endothelial damage. These include (i) endothelial cell expression of thrombomodulin (Th), which binds and inactivates thrombin, and of endothelial protein C receptor (EPCR) which binds and activates protein C. Protein C and protein S reduce thrombotic potential by inactivating thrombin, Va, and VIIIa; (ii) inherent antithrombin III (ATIII) expression, which also binds and inactivates thrombin; and (iii) expression of tissue plasminogen activator (tPA) and urokinase (uPA) which cleave plasminogen to form plasmin. This anticoagulant pathway is further regulated by thrombin activatable fibrinolysis inhibitor (TAFI) and a_2_-antiplasmin that both inhibit plasmin-induced fibrinolysis. (**B**) Mechanism of APL-mediated coagulopathy. Excess annexin II, a tPA/uPA receptor and activator that is present on the surface of APL promyeloblasts, contributes to hyperfibrinolysis and drives hemorrhage. Unrestricted malignant promyelocyte production of TF, cancer procoagulant (contributes to Xa production), and proinflammatory cytokines (i.e., IL1β, TNFα) lead to abnormal levels of IIa, driving the development of a consumptive coagulopathy and leading to thrombosis and subsequent hemorrhage. (**C**) Impact of exogenous therapies on the coagulopathy of APL. ATRA, platelets, and fibrinogen are depicted in red highlighting, text, and bars. ATRA decreases the expression of annexin II quickly, ameliorating hyperfibrinolysis, and via differentiation induction, reduces the number of APL promyeloblasts overexpressing proinflammatory/prothrombotic factors. Proposed interventions not currently recommended for clinical use that may address components of the coagulopathy of APL are depicted in yellow boxes with green bars indicating their potential mechanisms. Figure courtesy of Gabriel Ghiaur, Bryan Hambley, and Ciprian Tomuleasa. Originally published in Frontiers in Medicine, August 2021 [20].

At physiologic baseline, a delicate balance between hemostatic mechanisms preventing blood loss and antithrombotic mechanisms preventing blood flow obstruction maintains blood flow homeostasis [19]. Figure 1 demonstrates the basis of this balance between the coagulation cascade and its antithrombotic counterpart [20]. Tissue factor (TF) along with platelets are understood to be essential drivers of both primary and secondary hemostasis, leading to the formation of an initial platelet plug and a subsequent reinforcement of this plug via the clotting cascade/thrombin-driven production of fibrin to form a stable clot [21,22,23]. This pathway is counterbalanced by the activation of pathways leading to the production of plasmin which degrades fibrinogen/fibrin to prevent excess coagulation [21,22,23].

For the general treatment of bleeding diatheses, several agents are approved for clinical practice that act to stimulate or supplement components of the clotting cascade: tranexamic acid, recombinant FVIIa, prothrombin complex concentrate, and ε-aminocaproic acid [26,27,28]. Similarly, the number of antithrombotic therapies has rapidly expanded over recent decades and now includes warfarin, heparin and its derivatives enoxaparin and fondaparinux, and the direct-acting oral anticoagulants (DOACs), including direct thrombin inhibitors and direct factor Xa inhibitors [29]. Unfortunately, there has been minimal success in employing these advancements in APL treatment algorithms to reduce hemorrhagic and thrombotic complications [11].

Despite the body of research investigating the pathophysiology driving the coagulopathy of APL, adequate clinical data to inform the treatment and prevention of these complications are insufficient. Prior studies have attributed this to APL’s rare disease status, a lack of commercial support for clinical research in this disease, and/or the relative success of ATRA overshadowing the need for further improvements in care [20,30]. This review builds on this work to provide a unified document on the pathophysiology of hemorrhagic and thrombotic aberrations in APL, a synopsis of clinical parameters appropriate for risk-stratifying patients, an overview of the interventions commonly used in managing these complications, and an update on proposed novel approaches for mitigating coagulopathy in patients with APL.

## 2. Initial Presentation of APL

APL has a characteristic presentation with 80–90% of patients exhibiting various forms of severe mucocutaneous, intracerebral, or intrapulmonary hemorrhage and/or a thromboembolic event [15,31,32,33,34]. The diagnosis of APL is confirmed via histopathologic studies, and there are two common histologic variants: hypergranular and hypogranular [33]. The diagnosis of APL is commonly associated with laboratory values indicating a hyperinflammatory and coagulopathic state [31,32,33,35]. Although presenting symptoms were initially attributed to a disseminated intravascular coagulation (DIC)-like pathophysiology, it is now clear that the underlying mechanisms are more complex than a consumptive coagulopathy alone.

## 3. Pathophysiology of Hemorrhage in APL

Similar to DIC, a component of the coagulopathy of APL is driven by a clotting factor consumptive state that contributes to both hemorrhagic and thrombotic outcomes [35]. The translocation involving RARa that drives APL along with the proapoptotic/hyperinflammatory state leads to activation of the TF promoter [36]. In turn, the production and lysis of APL blasts containing large amounts of TF results in excess intravascular production of activated factor VII (VIIa) and subsequent prothrombotic cascade activity, further leading to excess fibrin production and clotting factor and fibrinogen depletion [37,38]. Consequently, it was previously assumed that the concomitant thrombotic and hemorrhagic phenotype of APL was akin to a sepsis-driven DIC; both present with pathologically low fibrinogen levels and an elevated prothrombin time (PT), elevated activated partial thromboplastin time (aPTT), and elevated levels of coagulation activation byproducts such as D-dimer [39]. However, the APL-driven coagulation cascade laboratory derangements significantly differ from other forms of DIC, as demonstrated by a frequent lack of abnormal levels of antithrombin III (ATIII) and protein C at presentation [40,41,42]. Moreover, the deviations in intrinsic coagulation pathway factor levels are less pronounced in APL patients as compared to sepsis-associated DIC, as shown by less-consistent aPTT and fibrinogen abnormalities [43]. Thus, it is clear that mechanisms beyond a consumptive coagulopathy contribute to the elevated rates of hemorrhage seen in APL.

One of the key differing mechanisms driving hemorrhage in APL is hyperfibrinolysis. Physiologic lysis of fibrin and fibrin-derived clots occurs via two major pathways. Primary fibrinolysis involves cleavage of plasminogen into its active form plasmin by tissue plasminogen activator (tPA) and urokinase (uPA) (Figure 1A). Secondary fibrinolysis is typically pathologic and seen in DIC. It is driven by exogenous clot breakdown in conditions of imbalanced activation of fibrinolytic enzymes and/or of inflammatory states leading to increased proteolytic susceptibility of fibrin [44]. Moreover, an increased ratio of plasmin to inhibitory α2-antiplasmin can promote hyperfibrinolysis [45,46]. APL does demonstrate components of secondary hyperfibrinolysis with decreased levels of PAI-1 leading to reductions in inhibitory α2-antiplasmin, as discussed further below [45,47]. However, primary hyperfibrinolysis is the dominant pathway driving hemorrhage (Figure 1B). Annexin II, the receptor and activator for tPA and uPA, is significantly overexpressed on the surface of promyeloblasts in APL [48,49]. This leads to excess annexin II-mediated enhancement of tPA/uPA-dependent conversion/activation of plasminogen to plasmin that normally serves to offset thrombosis, but in this case leads to increased hemorrhagic risk [50]. Interestingly, at baseline, there is elevated expression of annexin II in the central nervous system, and the further derangements in annexin II activity seen in APL may explain why intracerebral hemorrhage (ICH) makes up a majority of the overall APL-related bleeding deaths [51]. Moreover, multiple studies have demonstrated elevated plasmin, tPA, and uPA levels in APL, supporting the role of primary hyperfibrinolysis [30,41,48,52]. Along with elevated levels of uPA in APL, the uPA receptor is significantly overexpressed in both APL and AML, leading to further reinforcement of this pathway [53]. Thus, a major contributor to increased hemorrhagic events in APL is aberrant plasmin-driven fibrinolysis leading to an inability to form/maintain fibrin clots.

The physiologic pathway counteracting excess plasmin-mediated fibrinolysis involves regulatory compounds—such as plasminogen activation inhibitor 1 (PAI-1), alpha2-antiplasmin, and thrombin activatable fibrinolysis inhibitor (TAFI)—all acting to either reduce plasminogen activation or to directly inhibit plasmin-mediated fibrinogen and fibrin breakdown (Figure 1A). However, these critical regulatory pathways are also impaired in APL. A significantly diminished level of alpha2-antiplasmin is seen in APL, leading to an inability to counteract the APL-driven elevation in plasmin levels/activity [54]. Furthermore, multiple studies have shown that plasma PAI-1 activity and the formation of tPA/PAI-1 complexes were significantly reduced in APL patients via a proposed mechanism involving proteolytic degradation of PAI-1, leading to an inability to counteract APL-driven tPA hyperactivity [41,48]. Hence, there is a failure of the body’s physiologic mechanisms for regulating plasmin formation and preventing excess breakdown of fibrinogen/fibrin.

While a TF-driven consumptive coagulopathy and a hyperfibrinolytic state are likely the primary drivers of hemorrhage in APL, there is growing evidence to support other prohemorrhagic mechanisms. The first involves promyeloblast CD44 surface expression contributing to fibrin abnormalities [55]. A recent study of this mechanism in APL has demonstrated that membrane-bound CD44 binds and ligates fibrinogen, resulting in diminished fibrinogen levels and abnormal fibrin distribution [55]. Clots formed due to this CD44 fibrinogen interaction demonstrated resistance to fibrinolysis. CD44 was also shown to bind activated but not resting platelets via P-selectin, leading to excess platelet activation and consumption. In functional experiments, in vivo CD44 expression was associated with bleeding and wound healing complications in an APL mouse model [55]. While the mouse model indicated increased hemorrhagic events in this study, CD44 knockdown was also associated with a decreased bleeding time and increased fibrinogen, leaving some question as to the overall impact on coagulopathy in humans with APL [55]. This novel mechanism requires further investigation in APL patients as both a potential driver of hemorrhage and/or thrombotic events and as a potential molecular therapeutic target.

A second suggested mechanism contributing to hyperfibrinolysis in APL involves abnormal protease activity. Sepsis-driven DIC involves leukocyte elastase degradation of cross-linked fibrin outside of the physiologic plasmin-driven fibrin degradation pathway [56]. Moreover, high levels of leukocyte elastase are seen in APL, likely contributing to both hyperfibrinolysis and to proteolysis of tumor suppressor p200 CUX1, leading to disease progression [57,58]. However, a contradictory report demonstrated low levels of elastase degraded fibrin/fibrinogen in APL, raising a question about the role of elastase in contributing to fibrinolysis in APL [58]. A similar proteolytic mechanism involving matrix metalloproteases 2 and 9 (MMP2 and MMP9, respectively) has also been suggested. Elevated levels of MMP2/MMP9 are seen in non-APL patients with standalone ICH and ICH after cardioembolic stroke [59,60]. In one study of AML patients, high MMP2 levels were associated with significantly shorter survival time [61]. Interestingly, MMP9 expression levels correlated with increased blast crises in patients with AML and lower MMP9 expression correlated with improved survival [53,61]. Overall, while proteolytic enzyme abnormalities are associated with hyperfibrinolysis in AML and other malignancies, further characterization in APL is needed.

A final potential mechanism promoting hemorrhage in APL is a deficit in primary hemostasis. Most patients with APL are thrombocytopenic at the time of diagnosis, which may significantly contribute to early bleeding events. A study of platelet defects in APL demonstrated that while hemostatic variables improved during induction therapy, platelet counts remained significantly diminished until after four weeks of therapy [62]. On a platelet functional level, in a study comparing AML to immune thrombocytopenic purpura (ITP), flow cytometric measurements demonstrated morphologic platelet abnormalities indicative of an intrinsic dysfunction of platelet production and activity [63]. Moreover, this study and a more recent study comparing AML directly against healthy controls both found a significant reduction in the surface expression of platelet activation markers P-selectin and granulophysin, the platelet adhesion marker GP1b, and the platelet aggregation marker GPIIb-IIIa [63,64]. Such findings suggest potentially significant qualitative as well as quantitative platelet abnormalities contributing to elevated hemorrhage risk in APL and AML. However, these qualitative platelet defects need to be further studied exclusively in APL prior to making any definitive conclusions.

## 4. Hemorrhagic Events in APL

Early death in APL induction is commonly defined as death within 30 days of treatment induction [12,65]. Prior to the introduction of ATRA as a treatment modality, early death related to hemorrhage occurred in up to 26% of patients with APL [66,67]. Indeed, 3.5–10.2% of those with APM in two reports experienced early death prior to receiving any APL-directed therapy [13,68]. In a review of five large clinical trials that included ATRA in their induction regimens, early hemorrhagic death rates were as low as 3.7% and as high as 17.5% [11,13,43,69,70,71,72] (Table 1). Moreover, an analysis of APL outcomes across centers in California demonstrated that mortality at seven days post diagnosis decreased over time, with a rate of 19.8% from 1999–2002 to a rate of 13.7% from 2011–2014 [70]. Intracranial bleeding was the leading contributor to early death for both time frames [70]. Multiple studies have demonstrated similar findings that ICH was the most common subtype of hemorrhage contributing to early death (Table 1). Non-life-threatening bleeding at other sites, including mucosal membranes, the urinary tract, and the gastrointestinal tract, is also common [11,69,70,73].

Hemorrhagic death rates reported in the literature may be an underestimate of the true overall rate, as many patients not included in these studies present to hospitals with less capacity for the supportive care and therapy needed to achieve longer-term survival and confirmation of diagnosis. Of note, in one report of 963 APL patients treated at hospitals in California only 13.2% were treated at an NCI-CC, and treatment at a non-NCI-CC was associated with increased 30-day mortality [70]. Moreover, the most critical window for risk of hemorrhagic death appears to be the first 5–7 days following diagnosis/treatment induction [13,20,72]. The high early risk of bleeding-related mortality and the low overall incidence of APL leading to delayed diagnosis may have contributed to one report, which found 35% of APL patients with early death had not received ATRA treatment [65]. These data underscore the importance of a common understanding of how APL presents and treatment algorithms ensuring patients with a suspected diagnosis of APL, if stable, are transferred to a center with expertise in treating acute leukemias.

## 5. Risk Stratification for Hemorrhage and Hemorrhagic Death

Although the Sanz score and other risk stratification parameters have been used for assessing overall APL disease risk, there is not a standardized means of hemorrhagic risk stratification in APL. Naymagon and Mascarenhas recently conducted a review of parameters as predictors of hemorrhage in APL [75]. Table 2 is an extrapolation of that work and correlates various parameters with the literature supporting their association with risk of severe hemorrhage and/or early hemorrhagic death, along with suggested cutoff values proposed by various reports.

The most universally suggested and supported parameter for determining risk of hemorrhage, overall disease severity, and overall risk of early death in APL is leukocytosis. Clinicians and clinical trials commonly employ a WBC cutoff of >10 × 10^9^/L at presentation to risk-stratify adult and pediatric patients [11,79]. Along with a similar metric of total WBC leukemic blast count, WBC count has recently been associated with risk of hemorrhage as well [69,85]. Leukocytosis has a stronger association with bleeding risk across studies than any other risk factor and is a clinical red flag for patients at high risk for hemorrhage.

Platelet concentration, fibrinogen, and other hemostatic parameters have been proposed as adjunctive predictors of hemorrhagic risk, but the literature supporting these factors is less consistent overall than for total WBC count. Low fibrinogen levels correlate with significant risk of hemorrhage, coinciding with the hyperfibrinolytic mechanism underlying the hemorrhagic diathesis of APL [6,35,62,76,80,81]. In a study of 109 patients with APL, the ratio of fibrin degradation products to fibrinogen level was the strongest indicator of severity of bleeding, which further supports hyperfibrinolysis as a mechanism driving bleeding events [86]. Similarly, thrombocytopenia was shown to correlate with risk of hemorrhage in multiple studies, supporting the contribution of qualitative and quantitative impairments in primary hemostasis to hemorrhage in APL [11,13,14,35,62]. Notably, some analyses have demonstrated no significant correlation between fibrinogen or platelet levels at diagnosis and risk of hemorrhage [15,69,79,87]. Prolonged PT interval and D-Dimer elevations were also associated with increased bleeding risk but in fewer studies than other parameters, and there is a lack of adequate evidence to suggest aPTT correlates with hemorrhagic risk at all in APL [6,13,72,77]. This discrepancy between laboratory findings typically associated with a consumptive coagulopathy and their role in predicting hemorrhage in APL may be due to two mechanisms: (1) there are multiple pathways contributing to hemorrhage in APL rather than a simple consumptive state; (2) immediate transfusion of blood products to stabilize patients as per APL treatment guidelines confounds the true relationship of these parameters and bleeding risk in APL.

Other non-hemostatic laboratory values are also associated with risk of bleeding. Multiple studies have shown lactate dehydrogenase (LDH) strongly correlates with hemorrhagic risk, likely secondary to lysis of leukemic cells, contributing both to elevated LDH and to release of factors altering physiologic hemostasis in APL [13,35,62,78,84]. Creatinine level is also shown to correlate with bleeding risk in multiple studies, despite an unknown relationship of creatinine to APL coagulopathy [11,13,15,78,80]. Lastly, an Eastern Cooperative Oncology Group (ECOG) performance status score of 3 or 4 along with age >55 has been correlated with risk of hemorrhage/hemorrhagic death across multiple studies [6,11,12,13,69]. This association between risk of hemorrhage and both a validated performance status score and age substantiates the importance of utilizing parameters that directly or indirectly represent physiologic reserve to survive hemorrhage along with markers directly associated with hemostatic instability for risk assessment in APL.

## 6. Pathophysiology of Hypercoagulability in APL

Thrombotic events in APL are less common than hemorrhage, but they are still a major contributor to morbidity and a minor contributor to mortality. Virchow’s triad (vascular stasis, endothelial damage, and a hypercoagulable state) drives pathophysiologic thrombosis. Accumulating evidence suggests that APL causes thrombotic events via vascular inflammation that leads to endothelial damage and induces a systemic hypercoagulable state via multiple mechanisms outlined below.

The primary mechanism driving thrombosis in APL is a consumptive coagulopathy that also contributes to the prohemorrhagic state outlined previously. APL promyeloblasts contain large amounts of tissue factor (TF) driven by aberrant RARa activation of the TF promotor [36,37,38]. Moreover, instability in APL cells drives a proapoptotic state, further contributing to both activation of the TF promoter and systemic release of TF into the blood which triggers activated factor VII-mediated initiation of the clotting cascade [36]. This is supported by elevated levels of coagulation activation byproducts, including D-dimer and thrombin-antithrombin complexes in APL [39,88]. This hyperactivation of the coagulation cascade may also contribute to clotting factor and fibrinogen depletion and the comorbid APL-associated prohemorrhagic state [37,38]. This pathway is similar to a sepsis-driven consumptive coagulopathy, as both present with pathologically low fibrinogen levels and elevated PT and aPTT times [39]. However, PT and aPTT elevations are less consistent in APL, and preserved levels of ATIII and protein C indicate antithrombotic checkpoints are still present, suggesting that other mechanisms also contribute to the elevated rates of thrombotic events [40,41,42].

In multiple cancer types, intravascular microparticles derived from cancer cell and endothelial cell membrane components, platelets, and monocyte remnants cause a hypercoagulable state via surface expression of tissue factor (normally found in the cell membrane of endothelial cells, monocytes, and APL promyelocytes) [30,89]. Multiple studies show that these TF-containing microparticles (TFMPs) are present in APL and are derived from APL promyeloblasts, making this another potential mediator of APL-related hypercoagulability [90,91,92]. Mechanistic experiments illustrate that TFMPs found in APL are associated with elevated TF antigen levels and contribute both to increased thrombin generation and overall inflammation-associated hypercoagulability [91,92,93]. However, TFMPs have also been shown to contain tPA, PAI-1, and annexin II, which suggests multiple potential roles in the coagulopathy of APL [39]. The current studies investigating TFMPs in APL are limited in number and sample size, and the role of TFMPs in the coagulopathy of APL requires further investigation to gain an understanding of its interplay with other more defined mechanisms.

Another contributing driver of hypercoagulability in APL involves cancer procoagulant, a cysteine proteinase that activates factor X without needing factor VII [37]. As shown in Figure 1B, cancer procoagulant leads to direct activation of the common pathway of the coagulation cascade, resulting in thrombin activation and an increased propensity for the formation of fibrin clots [37]. Moreover, elevations of cancer procoagulant have been found in acute leukemia cells and especially in patients with APL [37,39,40]. The extent of this pathway’s contribution in the prothrombotic state associated with APL remains unclear.

A final etiology contributing to the prothrombotic state of APL is the cancer-driven release of inflammatory cytokines that cause vascular damage and upregulation of multiple components of the coagulation cascade. The cytokines IL-1B, IL-6, and TNF-α are overexpressed in patients with AML and APL [93,94,95]. These proinflammatory cytokines further drive excess endothelial cell TF expression, and TNF-α also decreases thrombomodulin transcription, leading to hypercoagulability and impaired regulation of coagulation [94,95,96,97,98]. Notably, targeting reduced levels of thrombomodulin with recombinant human thrombomodulin (rTM) to stabilize the coagulopathy of APL has been reported in Japan; early-phase clinical trials have shown it corrects aspects of coagulopathy in some patients with APL [99,100,101,102]. However, the efficacy and safety data supporting utilizing rTM for APL are not yet sufficient to recommend its use in routine care [103].

## 7. Characteristics of Thrombotic Events in APL and Markers of Thrombotic Risk

Despite improvements in APL outcomes, early thrombotic events still contribute to APL-associated morbidity (Table 3). Deep vein thrombosis (DVT), catheter-associated DVT, and arterial thromboembolic events make up the majority of thrombotic events. Thrombotic events, like hemorrhage, are most common early in the disease process, with >80% of thrombotic events occurring before or during induction treatment [18]. The overall incidence of thrombosis in APL varies from 5.1–24.5% in major studies quantifying these events [72,72,104,105]. Notably, there is no difference in incidence of thrombosis between older reports with chemotherapy-heavy treatment regimens and more recent studies utilizing treatment paradigms that include ATRA and ATO (Table 3).

Evidenced-based risk factors are less well defined for thrombosis compared with hemorrhage. A recent study of 84 patients with high-risk APL (WBC > 10 × 10^9^/L) demonstrated that elevated WBC/D-dimer ratio and reduced D-dimer/fibrinogen ratio were independent predictors of thrombosis [72]. Moreover, an elevated WBC/D-dimer ratio was much more elevated in patients with thrombosis as compared to patients with hemorrhage, and this may be a potential marker for distinguishing between hemorrhage and thrombosis risk [72]. In another report of 124 patients with APL, higher WBC count was associated with increased risk for thrombosis [17]. While leukocytosis has the most literature supporting an association with increased thrombotic events, there are not yet any adequately powered studies of thrombosis in APL to definitively establish additional risk factors for thrombosis.

## 8. Interaction between Induction Therapy and Coagulopathy in APL

Historically, treatment of APL utilized chemotherapeutic induction regimens involving anthracycline and cytarabine-based cytotoxic chemotherapy cycles. However, patients with APL had high rates of relapse and bleeding complications with cytotoxic chemotherapy alone [32,108]. ATRA was first demonstrated as a therapeutic agent in 1988 [109]. The addition of ATRA led to improvements in long-term remission and survival [108,110]. Thus, these initial efforts led to the incorporation of ATRA into first-line treatment regimens along with chemotherapy. Lo-Coco and colleagues then demonstrated that the combination of arsenic trioxide (ATO) with ATRA led to significant improvements in disease-free survival, with less toxicity than ATRA + chemotherapy in low- and intermediate-risk APL populations [8]. Importantly, ATO demonstrated a significantly reduced early death rate compared to non-ATO regimens, mitigating the toxicity burden during the highest-risk time for hemorrhage and thrombosis [111].

As the cornerstone of APL therapy, the mechanism of how ATRA stabilizes the coagulopathy of APL has been thoroughly investigated, and its effect on the coagulation cascade is summarized in Figure 1C. ATRA reduces annexin II expression, mitigating a primary component of the hyperfibrinolysis mechanism underlying hemorrhage [112]. This is coupled with an ATRA-induced upregulation of thrombomodulin that stabilizes the clotting cascade by preventing excess fibrin production and subsequent fibrinolysis [38]. Moreover, because ATRA promotes APL promyeloblast differentiation, it leads to decreased RARa-driven expression of TF and decreased production of cancer procoagulant, further diminishing the procoagulant activity of APL [37,38,113,114]. These mechanisms lead to a rapid stabilization of the dynamic coagulopathy of APL. This is reflected by the significant reduction of early morbidity and mortality and reduced coagulopathic events in APL following ATRA’s introduction as a treatment agent, and it underscores the importance of rapid initiation of ATRA at first suspicion of APL [103].

The impact of ATO on the coagulopathy of APL is less well understood compared with ATRA. ATO has been shown in vitro to downregulate tissue factor and cancer procoagulant factor; however, ATO has minimal effect on thrombomodulin [115]. Plus, the addition of ATO to APL treatment regimens was not associated with significant improvements in coagulopathy-associated markers such as platelet count, PT, fibrinogen levels, and/or D-dimer levels [116]. A phase 3 trial comparing an oral arsenic formulation with standard intravenous arsenic trioxide demonstrated no increase in grade 3–5 hemorrhagic events with oral arsenic, and none of the 66 patients treated with the oral arsenic formulation experienced a fatal hemorrhage [117]. Thus, there is some evidence that ATO may have more of a role in mitigating the hypercoagulable state associated with APL, but the role of ATO in addressing the hyperfibrinolytic and/or coagulation cascade abnormalities of APL requires further study.

Notably, it has recently been suggested that ATRA and ATO may transiently worsen APL coagulopathy due to their ability to provoke a cell death pathway [118]. This pathway involves the release of nuclear chromatins that form neutrophil extracellular traps (NETs) that are physiologically utilized by neutrophils in the innate immune response, a process termed ETosis [118]. ETosis results in endothelial cell damage and a procoagulable state [118]. Both ATRA (via increased TNF-α/IL-6 signaling and autophagosome activation) and ATO (via mTOR-dependent signaling) are associated with an induced ETosis [119,120,121]. The rapid ETosis after initiation of therapy may contribute to excess risk of thrombotic and hemorrhagic events [122,123]. ETosis may be an important mechanism underlying the persistence of hemorrhagic and thrombotic events during the first 2–3 weeks of APL treatment, despite the significant improvements in long-term outcomes seen with the introduction of ATRA and ATO.

A novel approach for mitigating the persistent coagulopathic risk and high mortality seen in APL treatment induction is the utilization of the anti-CD33 monoclonal antibody gemtuzumab ozogamicin (GO) in lieu of cytotoxic chemotherapy in high-risk APL [124]. Three studies evaluating GO in patients with high-risk APL showed excellent long-term disease control and less toxicity than chemotherapy-based regimens [125,126,127]. Further investigation into the impact of GO on APL-associated coagulopathy and overall bleeding/thrombosis rates is needed.

## 9. Current Strategies to Prevent and Treat Coagulopathy

Consensus guidelines for the management of APL were first published in 2009 by an expert panel on behalf of the European LeukemiaNet [128]. The relatively recent development of a standardized treatment algorithm highlights the challenges of providing evidence-based guidelines for a disease with a low incidence and high mortality if it is not recognized and treated. These guidelines were revised and expanded in 2019 to include data derived from an expanding body of APL research and to provide further clarification on treating the coagulopathy associated with APL [103]. The current recommendations for prevention and treatment of coagulopathic adverse outcomes seen upon presentation and during treatment induction are summarized in Table 4.

The mainstay of addressing the coagulopathy of APL involves the immediate initiation of ATRA along with supportive transfusion of fibrinogen concentrate/cryoprecipitate, platelets, and/or FFP as soon as APL is suspected. To prevent delays in treatment, the bedside clinical team and blood bank should be alerted that an intensive supportive transfusion regimen may be needed multiple times per day for the first 3–5 days of treatment. Moreover, invasive procedures such as central venous catheter placement or lumbar puncture should be avoided early in the disease course to avoid hemorrhagic complications of these procedures [103]. The proposed therapeutic mechanisms of common anticoagulants/antifibrinolytic options in patients with APL-induced coagulopathy are summarized in Figure 1C, and the current data regarding their usage in patients with APL are detailed below.

Heparin has been utilized to mitigate the consumptive coagulopathy of DIC in sepsis and has been reported in patients with APL. Heparin inhibits fibrinogenesis via activation of antithrombin III, leading to inhibition of factors II and X [129]. However, in a retrospective study of 268 APL patients, heparin was used in 94 patients, and no difference was seen between groups in early hemorrhagic death [34]. Moreover, heparin has been shown to increase transfusion requirements and exacerbate rates of delayed bleeding without clear benefit for reducing thrombotic and/or hemorrhagic events in APL [130]. Thus, heparin should not be routinely used in APL due to the risks of hemorrhage without clear clinical benefit [103].

Thrombomodulin, an anticoagulant normally expressed by endothelial cells that binds thrombin—leading to protein C activation and cleavage of factors V and VIII—has shown promise in stabilizing the coagulopathy of APL in limited retrospective studies [131]. Recombinant human thrombomodulin (rTM) has been investigated and utilized for addressing DIC in sepsis and other clinical scenarios [132]. It is also commonly utilized in Japan for patients with coagulopathy during APL induction [99,100,101,102]. The proposed mechanism involves stabilization of the coagulation cascade via protein C activation to prevent aberrant prothrombotic clotting factor activity and an accompanying downregulation of annexin II, leading to reduced plasmin overactivity and an enhancement of ATRA’s antihyperfibrinolysis mechanism [99]. A phase III randomized, double-blind clinical trial compared the use of heparin vs. rTM in the treatment of DIC due to hematologic malignancy or sepsis, and rTM alone showed a higher rate of DIC resolution than heparin alone (66.1% vs. 49.9%) and a significant improvement in clinical bleeding [133]. A retrospective surveillance safety study where 49 patients with APL received rTM prior to treatment induction demonstrated a 2% (1/49) rate of hemorrhagic early death, much lower than the current rate of 3.7–17.5% seen in the literature [102]. Larger phase III trials are needed to establish the efficacy and safety profile of rTM in APL.

The use of antifibrinolytic therapy is not recommended outside of clinical trials due to a lack of adequately powered, randomized-controlled clinical trials [103]. A retrospective study of 268 APL patients, with 67 receiving prophylactic antifibrinolytic therapy (tranexamic acid, epsilon-aminocaproic acid, or aprotinin), demonstrated no significant difference in incidence of early hemorrhagic death and/or complete remission rates compared to supportive care alone [34]. Moreover, a comparison of the PETHEMA study LPA99 protocol that utilized prophylactic tranexamic acid to the LPA96 protocol that did not showed no significant difference in early death or hemorrhagic events [15,134]. A potential future area of study could evaluate functional coagulopathy testing, such as thromboelastography, to identify patients undergoing acute hyperfibrinolysis and evaluate whether they could acutely benefit from antifibrinolytic therapy during specific periods of their course [20].

Another agent that has been reported, mostly in the setting of APL patients with severe intracerebral hemorrhage, is recombinant Factor VIIa (rFVIIa). Multiple case reports/case series document the use of rFVIIa in treating life-threatening bleeding, mainly ICH, in patients with APL [135,136,137]. One study demonstrated clinically significant management of severe bleeding in 5/7 (71%) patients with APL with rFVIIa [136]. However, the data are not robust, and there is a significant risk of thrombotic events associated with the use of rFVIIa [138,139]. Thus, while rFVIIa can be considered in immediately life-threatening bleeding, more research should be done to define whether the benefits outweigh the risk of utilizing rFVIIa in APL.

While less common than hemorrhage, thrombotic events still contribute to early morbidity and mortality in APL. Many targeted antithrombotic agents are utilized in other scenarios that warrant consideration for use in APL. The first is recombinant antithrombin III (rAT-III). Antithrombin–thrombin complexes are associated with active intravascular coagulation, and levels are significantly increased in APL [42,88]. rAT-III use in sepsis has had mixed results, and rAT-III use in APL has been associated with increased bleeding rates, especially when used in conjunction with heparin [140,141]. Thus, it has not been investigated further in APL and is not recommended for use.

Free tissue factor pathway inhibitor (TFPI) is elevated in patients with APL, and free TFPI inhibits activated factor X and factor VIIa-TF complexes [88]. The use of recombinant human TFPI (rhTFPI) in a murine APL model significantly decreased thrombin–antithrombin complexes and led to a stabilization of the APL-driven coagulopathy [88]. Large clinical studies using TFPI in patients with APL or other hematologic malignancies have not been reported.

A final potential avenue for addressing the prothrombotic diathesis of APL is recombinant human protein C (drotrecogin alfa). Physiologically, protein C regulates excess thrombosis, reduces inflammation, and promotes fibrinolysis [142]. Protein C levels in APL are variable [40]. Standard clinical use of drotrecogin alfa stopped after an increased risk of serious bleeding was noted in trials for patients with sepsis and DIC [143]. This increase in hemorrhage was found across multiple studies and led to the withdrawal of recombinant human protein C from the market in the United States for any disease [144]. Thus, although an interesting therapeutic mechanism, recombinant protein C use in patients with APL is not recommended.

## 10. Conclusions and Future Directions

Despite advancements in treatment, APL remains a disease with high risk of early mortality due to a characteristic coagulopathy. In today’s clinical landscape, a final major hurdle for minimizing APL morbidity and mortality is its associated coagulopathy. Research efforts continue to elucidate the underlying mechanisms of this coagulopathy, identify predictive markers for patient risk stratification, and determine optimal preventative and therapeutic approaches.

It is now clear that the coagulopathy of APL is distinct from sepsis-induced DIC. Beyond a consumptive coagulopathy, hyperfibrinolysis and defects in primary hemostasis are important components of the predisposition towards hemorrhage. Thrombosis in APL is driven by aberrant activation of tissue factor along with cancer procoagulant and prothrombotic inflammatory cytokine expression.

Many studies have attempted to identify markers and/or scoring parameters to predict hemorrhagic and/or thrombotic risk, with the aim of improving patient monitoring throughout the pre- and post-induction periods. The aim is to promote optimal triaging of patients in need of intensive monitoring, expedited allocation of transfusion products, and earlier identification of patients who may require novel approaches to mitigate severe coagulopathic events. There is a need for a consensus multicenter retrospective approach to identify consensus markers for predicting hemorrhagic and/or thrombotic risk in APL.

The delicate balance between a prothrombotic and prohemorrhagic state in APL has made it challenging to combat complications of one without tipping the balance in favor of the other. Beyond the addition of ATRA and ATO, the only supported interventions for reducing the risk of coagulopathy include avoiding high-risk interventions/medications and aggressive blood product support (Table 4). Some agents, such as thrombomodulin, rFVIIa, and TFPI, merit further focused investigation in certain clinical situations in APL. While novel agents remain in the investigational phase, there are potential non-pharmacologic solutions that can be employed immediately to further improve outcomes in APL. These include the following:

1. Improve the early recognition of APL across all health care systems based on clinical symptoms and laboratory abnormalities, and, when possible, transfer patients to a center with experience in the care of individuals with acute leukemia
2. Risk-stratify patients utilizing common laboratory parameter cutoffs
3. Minimize the time to induction treatment, including ATRA at first suspicion of diagnosis of APL and supportive blood product transfusion

Future studies should focus on retrospective and/or prospective multicenter collaborations aimed at achieving adequate power to ensure relevant findings will have both statistical and clinical significance. At this time, the most critical areas of focus include the following: (1) developing a unified set of markers predicting hemorrhagic and thrombotic risk to be incorporated into current risk stratification tools and used for future clinical trials; (2) expanding the preventative treatment armamentarium to include modalities outside of blood product transfusion; and (3) identifying effective treatments for life-threatening bleeding events such as intracranial hemorrhage. With these initiatives, clinicians and researchers will be able to further improve long-term survival in this disease.

## Figures and Tables

**Table 1:** Early death and hemorrhagic death in APL. Summary of major studies since 2010 detailing rates of overall early death and/or early hemorrhagic death within 30 days of diagnosis/treatment initiation, with most studies typically reporting rates of individual hemorrhage subtypes. Numbers of events may not add up to total, given incomplete reporting or multiple sites of bleeding in the same patient. ^a^ Meta-analysis of clinical trials; ^b^ Only included patients with high-risk APL as characterized by WBC > 10 × 10^9^.

Study/Year	Incidence of Early Death	Incidence of Early Hemorrhagic Death	Types of Hemorrhage
Lehmann et al., 2011 [65]	28.5% (30/105)	11.4% (12/105)	Intracranial 11/12 Pulmonary 1/12
Park et al., 2011 [12]	17.3% (242/1400)	N/A	Not reported
McClellan et al., 2012 [74]	18.6% (13/70)	11.4% (8/70)	Intracranial 7/8 Pulmonary 1/8
Lehmann et al., 2017 [11]	25.1% (49/195)	11.3% (22/195)	Intracranial 21/22 Pulmonary 1/22
Xu et al., 2017 [13]	23.1% (49/212)	17.5% (37/212)	Not reported
Mantha et al., 2017 [69] ^a^	N/A	3.7% (37/995)	Intracranial 24/37 Pulmonary 12/37
Ho et al., 2018 [70]	16.7% (161/963)	9.0% (87/963)	Only reported on intracranial events
Silva et al., 2019 [71]	29.5% (18/61)	9.8% (6/61)	Intracranial 5/6 Pulmonary 1/6
Xiao et al., 2022 [72] ^b^	Not reported	Overall rate of bleeding events (15/83) Hemorrhagic death rate 8.4% (7/83)	Intracranial 8/15 Pulmonary 2/15 Gastrointestinal 2/15 Other 2/15

**Table 2:** Laboratory parameters correlating with increased risk of hemorrhage, early hemorrhagic death, and/or overall early death in APL. Review of the literature from 2006–2022 for parameters that correlated with an increased risk of early hemorrhagic morbidity/mortality and/or with overall risk of early death.

Parameter	Supporting Studies	Suggested Cutoff Value
Leukocytosis	[11,13,15,35,62,69,72,76,77,78,79,80]	>10 × 10^9^/L >20 × 10^9^/L [69]
Fibrinogen	[6,35,62,76,80,81]	<1600 mg/L [81] <1000 mg/L [6,82]
Thrombocytopenia	[11,13,14,35,62]	<5 × 10^9^/L [83] <30 × 10^9^/L [11]
Lactate Dehydrogenase	[13,35,62,78,84]	>700 U/L [84]
Creatinine	[11,13,15,78,80]	>90 umol/L (1.02 mg/dL) [11]
Performance Status	[6,13,69,76]	ECOG 3–4 [13,69]
PT	[6,13,72,77]	N/A
D-Dimer	[62,80]	>4 mg/L [80]
Age	[13,69]	>55 years old [11]
Circulating Promyeloblast Level	[69,85]	>1.00–2.59 × 10^9^/L [69,85]

**Table 3:** Summary of studies characterizing thrombosis in APL.

Study	Type of Thrombosis	Incidence of Thrombosis
De Stefano et al., 2005 [106]	DVT/PE 1 Intracranial 2	9.6% (3/31)
Montesinos et al., 2006 [104]	DVT 17/39 PE 5/39 Cardiac 4/39 Intracranial 10/39 Other 3/39	5.1% (39/759)
Breccia et al., 2007 [17]	DVT 5/11 Cardiac 4/11 Intracranial 2/11	8.9% (11/124)
Chang et al., 2013 [105]	DVT 5/10 Cardiac 1/10 Intracranial 5/10	7.9% (10/127) ^a^
Mitrovic et al., 2015 [16]	DVT 7/13 Cardiac 2/13 Intracranial 2/13 Other 2/13	5.1% (13/63)
Bai et al., 2019 [107] ^b^	DVT 2/6 Intracranial 4/6	18.2% (6/33)
Xiao et al., 2022 [72] ^b^	DVT 4/10 Cardiac 0/10 Intracerebral 5/10 Other 3/10	24.5% (12/83)
Rashidi et al., 2013 [18] ^c^	DVT 27/94 Cardiac 25/94 Intracranial 27/94	N/A

^a^ One patient had both a DVT and an intracranial thrombosis; ^b^ only included patients with high-risk APL as characterized by WBC > 10 × 10^9^/L; ^c^ literature review of thrombotic events in patients with APL.

**Table 4:** Consensus summary of current recommendations for managing the coagulopathy of APL as reviewed and outlined in the most recent APL clinical consensus guidelines published by Sanz et al., in 2019 and further defined by Naymagon and Mascarenhas in 2020 [75,103].

Agent/Procedure	Indication
ATRA	Immediate initiation if APL is suspected as a diagnosis
Cryoprecipitate/Fibrinogen Concentrate ^1^	Initiation if APL is suspected as a diagnosis and fibrinogen < 100–150 mg/dL
Platelets	Initiation if APL is suspected as a diagnosis and platelet count < 30–50 × 10^9^/L
Fresh Frozen Plasma (FFP)/Cryoprecipitate	Initiation if APL is suspected as a diagnosis and INR > 1.5
Heparin	Not recommended unless severe thrombotic events
Recombinant Thrombomodulin	DIC—used in Japan, not currently recommended for broad clinical practice pending additional trials
Antifibrinolytic/Anticoagulant Therapy	Not recommended outside clinical trials

^1^ Cryoprecipitate and fibrinogen concentrate are not approved/available in some countries. Most leukemia centers have access to one of these products. In centers without access to either, FFP can be used, though adult-sized patients will often need 4–8 conventional units for a clinically meaningful increase in fibrinogen levels.
